# Serial Hydrolysis for the Simultaneous Analysis of Catecholamines and Steroids in the Urine of Patients with Alopecia Areata

**DOI:** 10.3390/molecules26092734

**Published:** 2021-05-06

**Authors:** Yu-Ra Lee, Bark-Lynn Lew, Woo-Young Sim, Jongki Hong, Bong-Chul Chung

**Affiliations:** 1 Molecular Recognition Research Center, Korea Institute of Science and Technology, Seoul 02792, Korea; T16627@kist.re.kr; 2 KHU-KIST Department of Converging Science and Technology, Kyung Hee University, Seoul 02447, Korea; 3 Department of Dermatology, Kyung Hee University Hospital at Gangdong, Kyung Hee University, Seoul 05278, Korea; bellotte@hanmail.net (B.-L.L.); sim@khnmc.or.kr (W.-Y.S.); 4 College of Pharmacy, Kyung Hee University, Seoul 02447, Korea

**Keywords:** alopecia areata, catecholamine, metanephrine, steroid, liquid chromatography–tandem mass spectrometry

## Abstract

Catecholamines and steroids are well-known neurotransmitters and hormones that rapidly change the excitability of neurons. Alopecia areata is a disease for which the exact cause is unknown, but it is considered to be associated with stress, and so the simultaneous analysis of catecholamines and steroids is required for the diagnosis of alopecia areata. Thus, we herein report the simultaneous analysis of catecholamines and steroids bearing different functional groups for the first time, during which it was necessary to carry out a serial hydrolysis procedure. Following hydrolysis of the urine samples to produce the free forms from the urinary conjugates, ethyl acetate extractions were carried out, and chemical derivatization was performed using dansyl chloride to increase the sensitivity of the liquid chromatography–tandem mass spectrometry method. The matrix effects and recoveries of this analytical method were validated, giving values of 85.4–122.9% and 88.8–123.0%, respectively. In addition, the method accuracy and precision were assessed, giving values of 0.4–21.5% and 2.0–21.6% for the intra-day and inter-day precisions, respectively. This validated method was then applied to identify differences between patients with and without alopecia areata, wherein the metanephrine content was found to be significantly higher in the alopecia areata patient group. This quantitative profiling method can also be applied to steroid-dependent diseases, as well as catecholamine-related diseases.

## 1. Introduction

Alopecia areata is a relatively common disease that occurs in ~2% of total population, in which the hair falls out in a circular shape. Usually, alopecia areata occurs in one or two areas of the head, but in severe cases, it can occur in several places at the same time, as well as in the eyebrows and beard [1]. Alopecia is considered a type of autoimmune disease, but the exact cause remains unknown. Although stress is considered to be the main cause of alopecia, no clear causal relationship has been established, although it may be hereditary. Alopecia areata can occur in any age group and also in children [2]. As indicated above, it has been reported to be associated with emotional or physical stress [3], and trauma [4]. Alopecia areata is difficult to treat medically, but recent developments based on molecular mechanisms appear to have the potential to alleviate the symptoms [5].

The catecholamines are neurotransmitters of the central nervous system, and they affect the entire human body from the cardiovascular system to neuropsychiatric functions. In this context, the diagnosis of neuroendocrine diseases and the confirmation of physiological and pathological conditions can be carried out through the quantitative analysis of catecholamines present in biological samples [6,7]. The catecholamines are metabolites associated with short-term stress [8] and chronic stress, and it has been demonstrated that high levels of catecholamines are present in the urine excretions of patients living in a stressful environment [9]. We therefore considered that catecholamine levels could be used to diagnose alopecia areata, wherein the determination of compounds including metanephrine and normetanephrine could investigate whether catecholamine metabolism changes in a group of patients with alopecia areata.

Other metabolites that are associated with short-term stress are the steroids. More specifically, neurosteroids are hormones that rapidly affect the functioning of the central nervous system [10]. Short-term stress increases testosterone secretion from the testes [11], and progesterone levels also increase during stress episodes [12]. In this context, we previously performed the quantitative analysis of the main androgens, including testosterone, DHT, and epitestosterone in the urine of both male and female alopecia areata patients [13].

In the analysis of biological matrices, the sampling of urine is more straightforward than the sampling of blood, cerebrospinal fluid, or biological tissues. In general, metabolites present in the blood are excreted from the plasma into the urine, and so urine can be considered an appropriate matrix for metabolite analysis. However, it has been previously reported that for the measurement of urinary metanephrine and normetanephrine, an acid hydrolysis step is generally required to convert the sulfate-conjugated metabolites into their free metabolites [14]. Similarly, the measurement of urinary steroids is normally performed following enzymatic hydrolysis, which converts the conjugated forms of the metabolites into free metabolites [15]. Therefore, for the simultaneous analysis of urinary catecholamines, metanephrine, normetanephrine, and steroids, the incorporation of a hydrolysis process is essential. In this context, our group has previously reported the simultaneous analysis of prostaglandins and androgens from the urine of androgenic alopecia patients [16], and of polyamines and steroids from the serum of breast cancer patients [17]. Based on these recent studies, we herein attempted to investigate the simultaneous analysis of catecholamines and steroids. Several studies have demonstrated the analysis of each compound separately [18]. In this study, we performed simultaneous analyses of catecholamine and steroid groups to extensively investigate the metabolites related to stress. Notably, more compounds can thus be analyzed using fewer samples, as compared to extant analyses.

Thus, we herein report the development of a method for the simultaneous analysis of catecholamines and steroids, followed by a subsequent examination of its applicability to their determination in the urine of alopecia areata patients. This will constitute the first study involving the quantitative metabolic profiling of compounds bearing two different functional groups using the LC-MS/MS technique.

## 2. Results

### 2.1. Optimization of the Dansyl Chloride Derivatization Procedure

**Figure 1:**
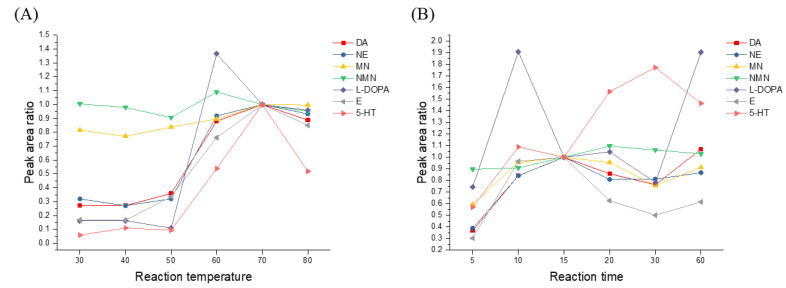
Optimization of the derivatization reaction conditions: (**A**) the reaction temperature, and (**B**) the reaction time (DA dopamine; NE: norepinephrine; MN: metanephrine; NMN: normetanephrine; L-DOPA: L-3,4-dihydroxyphenylalanine; E: epinephrine; and 5-HT: serotonin).

Catecholamines are low-molecular-weight primary amines in which one amino group is linked to the catechol ring. These amines are very polar substances and elute rapidly from analytical columns [19]. This low retention behavior of the catecholamines in the column, in addition to their low molecular weights, results in significant interference from the mobile phase, the sample matrix, and the environment in the low *m/z* region during MS analysis [20]. The use of dansyl chloride derivatization, which is generally suitable for all catecholamines, was therefore considered, with the aim of improving the analytical sensitivity [20]. The derivatization procedure employed herein was based on a previously reported literature method [21]. To confirm the optimal derivatization temperature, the effects of six reaction temperatures (30, 40, 50, 60, 70, 80 °C) using a fixed reaction time of 15 min were evaluated, and a reaction temperature of 70 °C was selected. Similarly, to confirm the optimal derivatization time, the reaction was carried out over times ranging from 5 min to 1 h (5, 10, 15, 20, 30, 60 min) using a fixed reaction temperature of 70 °C, and a reaction time of 15 min was found to be optimal. All experiments were repeated in triplicate, and averaged values are reported. As shown in Figure 1, seven catecholamines were subjected to the dansylated derivatization reaction to address the issues related to their low ionization efficiencies caused by the presence of a phenolic moiety.

### 2.2. Optimization of the Sample Preparation Procedure

In urine samples, the majority of catecholamines and steroids, in addition to normetanephrine and metanephrine, are excreted as their sulfate-conjugated or glucuronide-conjugated forms. Therefore, a deconjugation step is necessary for the simultaneous analysis of catecholamines and steroids. As mentioned above, due to the presence of different functional groups in these two classes of compounds, enzymatic hydrolysis is commonly employed for the steroids, while acidic hydrolysis is used for the catecholamines. In our study, when catecholamine deconjugation was performed using enzymatic hydrolysis, poor sensitivity and undetected catecholamine metabolites were present. Additionally, when steroid deconjugation was performed using acid hydrolysis, poor sensitivity and undetected steroid metabolites were present. We therefore performed serial hydrolysis using a β-glucuronidase/arylsulfatase solution (55 °C for 3 h) for deconjugation of the steroids and a 0.1 M HCl solution (90 °C for 30 min) for deconjugation of the catecholamines, normetanephrine, and metanephrine.

Following hydrolysis, a liquid–liquid extraction step was necessary to perform sample clean-up. However, due to differences in the structures between the two groups of compounds, selection of the appropriate extraction solvent was necessary. More specifically, steroids are non-polar, and so they extract well into non-polar solvents, such as methyl-tert-butyl ether and diethyl ether. However, the catecholamines are polar, and are not easily extracted with such solvents. We therefore examined two borderline polar aprotic solvents to allow the extraction of all compounds of interest, i.e., ethyl acetate and dichloromethane. However, it was found that the dichloromethane sank below the sample mixture, thereby rendering extraction difficult. As a result, we selected ethyl acetate for sample clean-up, and using this method, the separation of seven catecholamines and six steroids was successful without interference from any background peaks.

### 2.3. Liquid Chromatography–Tandem Mass Spectrometry

**Figure 2:**
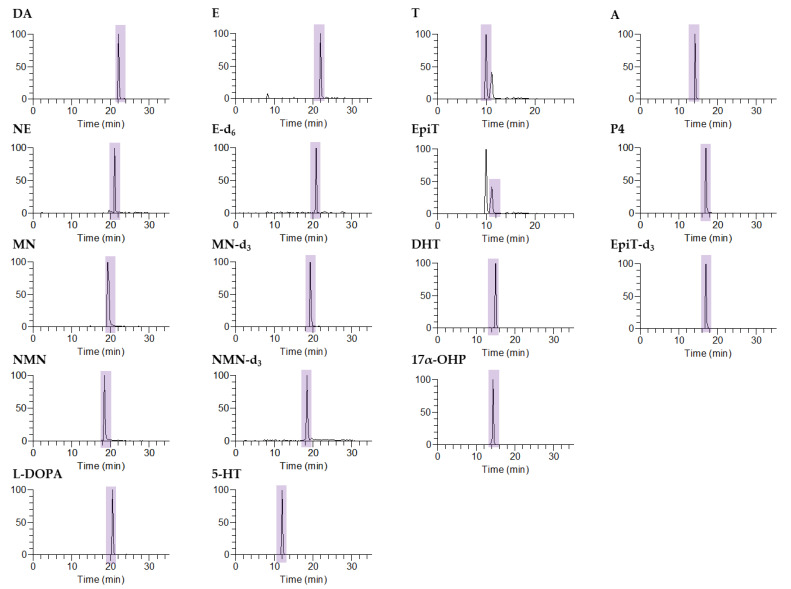
Chromatograms of seven catecholamines, six steroids, and four internal standards in the selective reaction monitoring (SRM) mode following pretreatment with the target standard (500 ng/mL). (DA: dopamine; NE: norepinephrine; MN: metanephrine; NMN: normetanephrine; L-DOPA: L-3,4-dihydroxyphenylalanine; E: epinephrine; E-d_6_: epinephrine-d_6_; MN-d_3_: metanephrine-d_3_; NMN-d_3_: normetanephrine-d_3_; 5-HT: serotonin; T: testosterone; EpiT: epitestosterone; DHT: dihydrotestosterone; 17α-OHP: 17α-hydroxyprogesterone; A: androstenedione; P4: progesterone; and EpiT-d_3_: epitestosterone-d_3_).

Catecholamines have a positive charge due to the presence of amino groups in their molecular structure, and so they easily bind with other substances. Thus, to facilitate the sensitive and accurate determination of the catecholamines, we performed a derivatization step using dansyl chloride, as mentioned above. Reaction of the dansyl chloride with the hydroxyl and amine functional groups produced higher-molecular-weight compounds, thereby allowing their selective extraction, as shown in Figure 2.

As outlined in Table 1, all catecholamines and steroids produced protonated precursor ions [M+H]^+^. The high sensitivity of this method was demonstrated by the detection of dopamine and L-DOPA as their [M + H − C_12_H_12_NO_2_S]^+^ ions, i.e., [M + H − 234]^+^, 5-HT as the [M + H − C_12_H_13_NO_3_S]^+^ ion, i.e., [M + H − 250]^+^ [22], and NE, NMN, MN, E, MN-d_3_, NMN-d_3_, E-d_6_, T, EpiT, A, P_4_, and 17α-OHP as their [M + H − H_2_O]^+^ ions, i.e., [M + H − 18]^+^. Similarly, DHT and EpiT-d_3_ were observed as their [M + H − 2H_2_O]^+^ ions, i.e., [M + H − 36]^+^.

### 2.4. Method Validation

For normalization, the ion peak area of each analyte was divided by that of the corresponding IS (i.e., catecholamine groups—E-d_6_; MN—MN-d_3_; NMN—NMN-d_3_; and steroid groups—EpiT-d_3_). The developed method was validated by determination of its limit of quantitation (LOQ), accuracy, and precision using quality control (QC) samples. Linear regression equations were obtained using eight concentrations of all analytes (i.e., 1, 5, 10, 50, 100, 500, 1000, and 5000 ng/mL, with the exception of L-DOPA, for which 1, 5, and 10 ng/mL were excluded, but 20, 200, and 2000 ng/mL were used instead). LOQ values of 1 ng/mL were determined for the majority of analytes, although a higher value (i.e., 20 ng/mL) was determined for L-DOPA. We also performed matrix effect and recovery tests; no significant matrix effects were observed for any of the analytes (85.4–122.9%), and overall, good recoveries were obtained (i.e., 88.8–123.0%) (see Supplementary Materials for Table S1).

For an analysis of the catecholamines and steroids, the method accuracy and precision were evaluated using five QC samples of different concentrations (Table 2). The precision was determined based on the coefficient of variation (% CV), while the accuracy was evaluated based on the amount of analyte detected as a mean ± standard deviation (SD). For catecholamine analysis, the intra-day (*n* = 3) precisions (% CV) ranged from 1.3 to 19.2%, while the inter-day (*n* = 3) precision (% CV) ranged from 2.1 to 19.5%. For steroid analysis, the intra-day (*n* = 3) precisions (% CV) ranged from 0.4 to 21.5%, while the inter-day (*n* = 3) precision (% CV) ranged from 2.0 to 21.6%.

### 2.5. Application

To evaluate the potential application of the developed method, we analyzed the catecholamines and steroids present in the urine samples of alopecia areata patients and compared them with the levels detected in normal controls (Table 3).

Based on the results of a Student’s *t*-test, the levels of metanephrine were found to be significantly higher in patients suffering from alopecia areata. Although no studies exist on the correlation between alopecia areata and metanephrine, several papers have reported that the levels of metanephrine are significantly increased in depressed patients [23,24,25]. In addition, some reports have described a significant decrease in metanephrine levels during the psychological treatment of depressed patients [26]. Interestingly, increased urinary metanephrine excretion has been detected in dogs subjected to stressful environments [27]. In combination with these previous studies, our results therefore suggest that further research is required in the context of the pathogenesis of alopecia and its relationship with these higher metabolites. It should also be noted here that the catecholamine concentrations detected in the normal control subjects were similar to those reported in previous studies [28,29], and among the steroids analyzed, the androgen and progesterone levels were similar to those of reference ranges [30,31].

## 3. Materials and Methods

### 3.1. Chemicals and Materials

Seven catecholamines, six steroids, and four deuterium-labeled internal standards were used for the purpose of this study. The reference standards were obtained from Tokyo Chemical Industry (Tokyo, Japan) and Sigma-Aldrich (St. Louis, MO, USA). The internal standards (ISs), i.e., epitestosterone-d_3_ (EpiT-d_3_), normetanephrine-d_3_ (NMN-d_3_), metanephrine-d_3_ (MN-d_3_), and epinephrine-d_6_ (E-d_6_), were obtained from Sigma-Aldrich and CDN Isotopes (Quebec, Canada). All reagents employed for the preparation of the artificial urine (AU) and the derivatizing solutions were purchased from Sigma-Aldrich. Hydrochloric acid (HCl), sodium hydroxide (NaOH), sodium acetate, acetic acid, and formic acid were obtained Sigma-Aldrich. The β-glucuronidase/arylsulfatase enzyme hydrolysis solution derived from *Helix pomatia* was purchased from Roche (Basel, Switzerland). The solvents, namely ethyl acetate, acetonitrile, acetone, and methanol (MeOH), were obtained from Honeywell Burdick & Jackson (Muskegon, MI, USA). Prior to use, deionized water (DW) was filtered using a Milli-Q reference water purification system (Burlington, MA, USA).

### 3.2. Preparation of the Standard Solutions

The catecholamine standard solutions were prepared by dissolution in MeOH (except L-DOPA, which was prepared by dissolution in a 1 M HCl solution) to give a concentration of 1000 μg/mL, while the steroid standard solutions were prepared by dissolution in acetonitrile to give a concentration of 1000 μg/mL. Working solutions were obtained by the dilution of the standard solutions using the appropriate solvents to give concentrations ranging from 100 μg/mL to 1 ng/mL. The ISs for catecholamine normalization (i.e., containing metanephrine-d_3_, normetanephrine-d_3_, and epinephrine-d_6_) were prepared by dissolution in MeOH to give a concentration of 100 μg/mL. Working solutions were prepared by dilution in methanol to give a concentration of 1 μg/mL. In addition, the EpiT-d_3_ IS was prepared by dissolution in acetonitrile to give a concentration of 10 μg/mL, and the corresponding working solution was prepared by dilution in acetonitrile to give a concentration of 1 μg/mL. All solutions were stored at −20 °C until required for use.

### 3.3. Liquid Chromatography–Tandem Mass Spectrometry

The urinary catecholamines, normetanephrines, metanephrines, and steroids were analyzed using a Shiseido nanospace SI-2 (Osaka, Japan) liquid chromatograph combined with a Thermo LTQ XL mass spectrometer (San Jose, CA, USA). The column employed for analysis was a C18 Hypersil Gold column (3 µm, 2.1 × 150 mm), and the mobile phase consisted of mobile phase A (0.1% formic acid in 95% DW) and mobile phase B (0.1% formic acid in 95% acetonitrile) at a flow rate of 200 µL/min at 35 °C. A gradient elution mode was employed as follows: 0 min, 12% B; 0–17 min, 12–88% B; 17–25 min, 88% B; 25–28 min, 88–12% B; and 28–35 min, 12% B.

### 3.4. Sample Preparation

**Figure 3:**
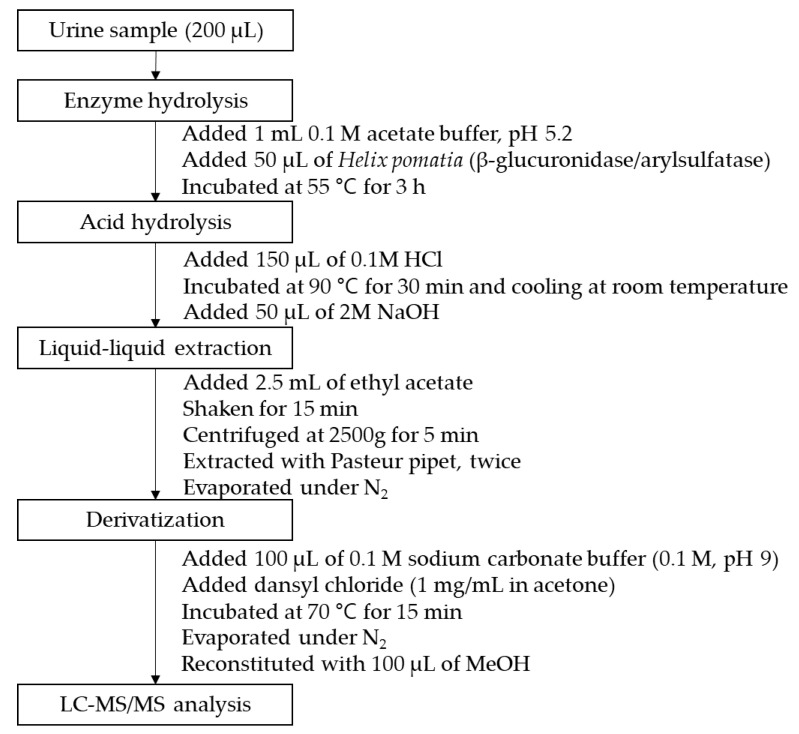
Schematic outline of the sample preparation procedure.

A urine volume of 200 μL was used for sample preparation. Aliquots (50 μL) of the IS (1 μg/mL), the β-glucuronidase/arylsulfatase solution (50 μL), and the acetate buffer (1 mL) were then added to the urine sample and the resulting mixture was heated for 3 h at 55 °C. Acid hydrolysis was then carried out by the addition of a 0.1 M HCl solution (100 μL) and subsequent heating at 90 °C for 30 min. After this time, the mixture was cooled, and a 2 M NaOH solution (50 μL) was added to adjust the pH to 11. The mixed solution was then shaken for 15 min in the presence of ethyl acetate (2.5 mL) and layered using a centrifuge. This extraction procedure was performed twice, after which the combined supernatant was transferred to a new tube and evaporated under a flow of nitrogen. For the subsequent derivatization step, dansyl chloride (1 mg/mL solution in acetone, 100 µL) and sodium bicarbonate buffer (0.1 M, 100 µL) were added, and the resulting mixture was stirred for 15 min at 70 °C. After the evaporation of the reaction solvent under a flow of nitrogen, the residue was reconstituted with MeOH (100 µL) and analyzed in the selected reaction monitoring (SRM) mode. Figure 3 outlines the overall experimental procedure.

### 3.5. Sample Information

The urine samples employed in this study were obtained from 29 normal controls (aged 20–45, mean age 31.21) and 40 alopecia areata patients (aged 10–64, mean age 39.35). The urine samples were collected from the Department of Dermatology, Kyung Hee University Hospital (Gang-dong). Written consent was obtained from all participants. Moreover, written consent for minors, i.e., those aged under 18 years, was obtained from a guardian in all cases. The study was approved by the University of Kyung Hee’s Ethics Committee (IRB No. 2016-11-037-007).

### 3.6. Validation

The calibration solution and quality control solution for method validation were prepared by adding a working solution obtained by the continuous dilution of each standard solution to the AU, which was produced according to a previously described method [32]. Quality control samples were prepared at five different concentrations on each validation day.

Standard solutions for the calibration curve were prepared at concentrations of 1, 10, 50, 100, 500, 1000, 2000, and 5000 ng/mL for each solution. The linearity was evaluated using the Pearson correlation coefficient (R^2^), while the limit of quantitation (LOQ) was confirmed based on a signal-to-noise (S/N) ratio ≥ 10. The recovery test was performed to confirm how the amount of the analyte changed during the pretreatment process. All peak areas corresponding to samples that had undergone the pretreatment process and all peak areas corresponding to samples that had undergone derivatization were confirmed. In addition, we evaluated any potential matrix effects using the following relationship between the signals: ratio of the spiked target and IS solutions in the urine samples—ratio of the target and IS solutions in the AU samples)/(ratio of the corresponding target and IS solutions) × 100 (%) [7]. The method accuracy and precision were confirmed using QC samples at five different concentrations. More specifically, the accuracy was evaluated from the amount of compound detected (mean ± standard deviation (SD)), while the precision was determined using the coefficient of variation (% CV). The intra- and inter-day validations were performed by analyzing three replicate samples and samples obtained on three different days, respectively.

### 3.7. Creatinine Measurement

The creatinine concentrations in the urine samples were determined using a QuantiChrom™ creatinine analysis kit (based on the Jaffe reaction) and a BioAssay System UV–vis spectrophotometer. More specifically, the urine samples were centrifuged for 5 min to allow protein precipitation, and were then combined with a 1:1 (*v/v*) mixture of Reagent A and Reagent B. An aliquot (500 mg/dL, 15 μL) of the creatinine standard solution was then added to a cuvette and mixed with the working reagent (1 mL). The optical density was measured immediately (OD_STD0_) and after 5 min (OD_STD5_) at 490–530 nm. Subsequently, an aliquot of the urine sample (15 μL) was added to a cuvette and mixed with the working reagent (1 mL). Similarly, the optical density was measured immediately (OD_SAMPLE0_) and after 5 min (OD_SAMPLE5_) at 490–530 nm.

## 4. Conclusions

In this study, we performed a simultaneous analysis of catecholamines and steroids from the urine of patients suffering from alopecia areata. Due to the fact that catecholamines have low molecular weights, it was necessary to derivatize the catecholamines using dansyl chloride to increase the sensitivity of the analytical method. In addition, a serial hydrolysis process was employed for all urine samples, which was based on enzymatic hydrolysis and subsequent acid hydrolysis. Following extraction and derivatization, analysis was performed using liquid chromatography–tandem mass spectrometry. Using this method, we confirmed that seven catecholamines and six steroids were successfully extracted and detected from the urine samples, without any interference being observed from other peaks. Based on our results, we revealed the differences in the metabolic levels of these compounds between normal control and patients suffering from alopecia areata. We therefore expect that our method will reduce analytical costs and reduce analysis times through the combined determination of two groups of metabolites using the same sample pretreatment and analysis procedure. In addition, it should be noted that our method is applicable to both catecholamine-related diseases (e.g., neurodegenerative diseases) and steroid-related diseases, such as androgenic alopecia.

## Figures and Tables

**Table 1:** Optimized MS information for the simultaneous analysis of catecholamines and steroids.

Compound	Abbrev.	Precursor Ion (*m*/*z*)	Product Ion (*m*/*z*)	Normalized Collision Energy (%)	Retention Time (min)
*Catecholamines*					
Dopamine	DA	853	619	39	22.1
Norepinephrine	NE	869	851	32	21
Normetanephrine	NMN	650	632	30	18.1
Metanephrine	MN	664	646	31	19.2
L-3,4-dihydroxyphenylalanine	L-DOPA	897	663	30	20.5
Epinephrine	E	883	865	27	21.9
Serotonin	5-HT	410	160	25	11.9
*Steroids*					
Testosterone	T	289.2	271.3	56	13.4
Dihydrotestosterone	DHT	291.2	255.3	24	14.9
Epitestosterone	EpiT	289.2	271.3	26	14
Androstenedione	A	287.2	269.3	48	13.8
Progesterone	P4	315.2	297.3	30	16.6
17α-Hydroxyprogesterone	17α-OHP	331.2	313.3	22	13.6
*Internal standards*					
Metanephrine-d_3_	MN-d_3_	667	649	27	19.2
Normetanephrine-d_3_	NMN-d_3_	653	635	25	18.1
Epinephrine-d_6_	E-d_6_	889	871	31	20.7
Epitestosterone-d_3_	EpiT-d_3_	292.2	256.4	35	14.6

**Table 2:** Accuracies and precisions of the method for the various analytes based on intra- and inter-day assays.

Analytes	Spiked Concentration (ng/mL)	Intra-Day Assay		Inter-Day Assay	
		Amount Found (Mean ± SD)	% CV	Amount Found (Mean ± SD)	% CV
DA	10	10.88 ± 0.66	6	10.27 ± 1.72	16.7
	50	51.39 ± 9.85	19.2	53.04 ± 6.8	12.8
	100	102.25 ± 8.4	8.2	89.92 ± 7.26	8.1
	500	520.75 ± 75.44	14.5	454.32 ± 70.47	15.5
	5000	5202.45 ± 122.27	2.4	4919.39 ± 393.18	8
NE	10	8.43 ± 0.67	8	11.25 ± 0.7	6.2
	50	49.03 ± 3.18	6.5	51.31 ± 5.17	10.1
	100	96.86 ± 14.78	15.3	100.83 ± 11.57	11.5
	500	503.66 ± 85.16	16.9	439.01 ± 32.12	7.3
	5000	5426.45 ± 69.68	1.3	4733.22 ± 532.58	11.3
MN	10	10.53 ± 1.22	11.6	9.01 ± 0.49	5.5
	50	53.01 ± 6.51	12.3	54.11 ± 5.73	10.6
	100	95.77 ± 17	17.8	95.6 ± 11.06	11.6
	500	508.78 ± 58.68	11.5	440.56 ± 54.75	12.4
	5000	4774.86 ± 103.34	2.2	4863 ± 103.34	2.1
NMN	10	10.32 ± 0.38	3.7	10.24 ± 1.07	10.5
	50	45.79 ± 3.14	6.9	51 ± 7.04	13.8
	100	99.69 ± 15.36	15.4	95.12 ± 8.21	8.6
	500	519.83 ± 75.53	14.5	399.37 ± 24.48	6.1
	5000	4652.22 ± 507.11	10.9	5265.81 ± 885.64	16.8
L-DOPA	50	50.5 ± 8.18	16.2	58.1 ± 1.47	2.5
	100	97 ± 3.71	3.8	103.71 ± 10.52	10.1
	500	532.9 ± 60.88	11.4	501.39 ± 59.99	12
	5000	5483.72 ± 232.77	4.2	4204.8 ± 346.51	8.2
E	10	10.52 ± 1.32	12.5	8.51 ± 0.66	7.7
	50	55.47 ± 8.85	16	46.82 ± 8.09	17.3
	100	91.65 ± 11.73	12.8	105.4 ± 20.52	19.5
	500	427.72 ± 20.21	4.7	436.41 ± 15.01	3.4
	5000	4767.63 ± 300.46	6.3	4961.5 ± 390.27	7.9
5-HT	10	9.37 ± 0.84	9	10.03 ± 1.29	12.8
	50	43.56 ± 2.48	5.7	48.64 ± 4.73	9.7
	100	103.13 ± 12.04	11.7	98.7 ± 3.19	3.2
	500	523.62 ± 57.83	11	506.1 ± 80.12	15.8
	5000	5278.65 ± 345.51	6.6	5390.38 ± 723.93	13.4
T	10	10.59 ± 1.18	11.1	10.41 ± 0.32	3.1
	50	48.34 ± 5.61	11.6	52.53 ± 6.95	13.2
	100	104.83 ± 8.02	7.7	97.14 ± 5.03	5.2
	500	474.64 ± 31.72	6.7	473.68 ± 28.46	6
	5000	4185.64 ± 129.65	3.1	4643.86 ± 210.06	4.5
EpiT	10	9.66 ± 0.35	3.6	11.56 ± 1.7	14.7
	50	46.56 ± 6.46	13.9	52.8 ± 6.16	11.7
	100	99.27 ± 16.46	16.6	82.6 ± 13.4	16.2
	500	465.64 ± 59.24	12.7	504.23 ± 85.7	17
	5000	5738.34 ± 24.61	0.4	5116.56 ± 790.31	15.5
DHT	10	9.79 ± 0.44	4.5	10.35 ± 0.23	2.3
	50	47.93 ± 6.02	12.6	47.45 ± 6.83	14.4
	100	105.11 ± 6.83	6.5	101 ± 1.97	2
	500	505.37 ± 91.54	18.1	484.7 ± 32.76	6.8
	5000	5181.97 ± 1111.79	21.5	5655.49 ± 236.7	4.2
17α-OHP	10	10.25 ± 1.47	14.4	10.56 ± 1.04	9.9
	50	51.04 ± 7.45	14.6	50.53 ± 2.93	5.8
	100	98.82 ± 6.47	6.6	86.37 ± 2.88	3.3
	500	426.97 ± 23.82	5.6	500.5 ± 72.76	14.5
	5000	5058.79 ± 960.5	19	4784.76 ± 1032.4	21.6
A	10	10.12 ± 1.49	14.7	9.21 ± 0.7	7.7
	50	54.6 ± 7.59	13.9	49.93 ± 8.19	16.4
	100	111.76 ± 3.53	3.2	112.89 ± 14.08	12.5
	500	528.46 ± 65.34	12.4	468.24 ± 51.44	11
	5000	5247.42 ± 558.88	10.7	4634.58 ± 475.97	10.3
P4	10	9.25 ± 0.77	8.3	9.49 ± 0.81	8.5
	50	52.23 ± 9.67	18.5	58.33 ± 3.22	5.5
	100	103.54 ± 13.58	13.1	100.94 ± 17.48	17.3
	500	433.69 ± 40.93	9.4	413.79 ± 14.05	3.4
	5000	4717.45 ± 645.9	13.7	5097.21 ± 501.55	9.8

**Table 3:** Urinary concentrations of seven catecholamines and six steroids in samples obtained from alopecia areata patients and normal controls (ng/mg creatinine).

	Normal Controls (*n* = 29)		Patients (*n* = 40)		*p* Value
	Mean ± SD	Range	Mean ± SD	Range	
DA	202.23 ± 132.6	31.26–507.96	147.47 ± 216.85	8.28–903.06	0.302
NE	125.88 ± 210.98	8.73–1052.7	115.9 ± 222.37	1.29–1261.24	0.856
MN	48.88 ± 115.67	3.87–612.13	134.72 ± 183.23	5.45–720	0.036
NMN	27.99 ± 50.52	2.1–239.32	81.43 ± 109.72	1.49–444.94	0.053
L-DOPA	236.64 ± 299.21	14.36–1437.92	222.46 ± 625.27	1.89–3530.4	0.914
E	29.44 ± 35.7	5.33–181.96	36.47 ± 72.21	1.04–296.97	0.644
5-HT	203.67 ± 259.88	6.51–1082.05	112.19 ± 156.61	2.57–574.75	0.317
T	42.16 ± 114.11	2.25–508.47	22.76 ± 43.8	1.94–243.06	0.397
EpiT	42.53 ± 76.91	4.04–410.4	24.69 ± 24.56	1.86–105.89	0.201
DHT	26.69 ± 41.71	2.06–171.75	15.75 ± 17.66	1.12–66.46	0.232
17α-OHP	12.29 ± 15.66	1.16–58.77	7.48 ± 6.45	1.22–33.13	0.117
A	10.27 ± 19.88	1.65–88.2	5.54 ± 5.77	1.25–22.65	0.284
P4	8.96 ± 8.28	1.75–31.17	13.42 ± 15.22	1.18–71.32	0.248

## Data Availability

The data presented in this study are openly available. Data available in a publicly-accessible repository.

## Supplementary Materials

The following are available online, Table S1: Calibration ranges, linear regressions, limits of quantitation, matric effect results, and recovery test results for the developed method for catecholamine and steroid determination.

## Sample Availability

The samples of compounds are not available from authors.
