# Supplementary material for: Serial Hydrolysis for the Simultaneous Analysis of Catecholamines and Steroids in the Urine of Patients with Alopecia Areata
Source: Molecules. 2021 May 6;26(9):2734. doi: 10.3390/molecules26092734 (PMC8125454; doi:10.3390/molecules26092734)
Supplement: Supplementary file 1 [file molecules-26-02734-s001.zip › molecules-1203927-supplementary.pdf]

SUPPLEMENTARY MATERIALS

Table S1. Calibration ranges, linear regressions, limits of quantitation, matrix effect results, and recovery test results for the developed method for catecholamine and steroid determination.

Analytes	Calibration range	Linear regression equation	R^2	LOQ (ng/mL)	Matrix effect (%)	Recovery test (%)
DA	1–5000	y=0.0004x-0.0117	0.9997	1	88.7	99.8
NE	1–5000	y=6E-05x+0.0017	0.9987	1	93.8	102.3
MN	1–5000	y=0.0022x-0.2176	0.9952	1	90.5	103.5
NMN	1–5000	y=0.0051x-0.511	0.9924	1	93.2	90.5
L-DOPA	20–5000	y=6E-06x+0.0047	0.9972	20	108	105.3
E	1–5000	y=2E-05x+0.0007	0.9985	1	112.4	115.3
5-HT	1–5000	y=0.0009x-0.087	0.9957	1	122.9	88.8
T	1–5000	y=0.0013x+0.0594	0.9973	1	85.4	101.9
EpiT	1–5000	y=0.0002x+0.0825	0.9951	1	95.4	92.3
DHT	1–5000	y=0.0013x-0.111	0.9965	1	96.6	123
17α-OHP	1–5000	y=0.0013x-0.1406	0.9909	1	107.3	102.4
A	1–5000	y=0.0004x-0.0522	0.9905	1	102.7	102.4
P4	1–5000	y=0.0009x-0.0672	0.9969	1	90	102.9
